# Potential of Sentinel-3 snow cover fraction data for improving hydrological simulations at the regional scale

**DOI:** 10.1038/s41598-026-46403-2

**Published:** 2026-03-30

**Authors:** Mitra Tanhapour, Juraj Parajka, Gabriele Schwaizer, Mariette Vreugdenhil, Silvia Kohnová, Kamila Hlavčová, Roman Výleta, Ján Szolgay

**Affiliations:** 1 Department of Land and Water Resources Management, Slovak University of Technology, Bratislava, 81005 Slovakia; 2 Centre for Water Resource Systems, TU Wien, Vienna, 1040 Austria; 3 Institute of Hydraulic Engineering and Water Resources Management, TU Wien, Vienna, 1040 Austria; 4 ENVEO-Environmental Earth Observation IT GmbH, Fürstenweg 176, Innsbruck, 6020 Austria; 5 Department of Geodesy and Geoinformation, TU Wien, Vienna, 1040 Austria

**Keywords:** Sentinel-3 Snow Cover Fraction, regional hydrologic modelling, multiple-objective calibration, overall accuracy at climate stations, Climate sciences, Environmental sciences, Hydrology

## Abstract

Satellite snow cover observations have been shown to enhance the calibration of conceptual hydrologic models. Recent advance in the mapping of snow cover fraction brings new satellite products and datasets. This study assesses the accuracy and potential of a newly developed snow cover fraction (SCF) product derived from Sentinel-3 observations. The product is developed using a physically based spectral unmixing approach that maps daily snow cover fractions at a 200 m spatial resolution over mountain regions. The main objective of this study is to evaluate the potential of the SCF for improving hydrological simulations at the regional scale. The specific aims are to compare the accuracy of snow cover mapping with daily snow depth observations at 631 climate stations and to assess and compare the runoff and snow model efficiencies obtained from multiple-objective calibration and calibration to runoff only. The analysis is performed using 188 lowland and alpine catchments in Austria. The results show that SCF agrees very well with snow depth observations at climate stations as documented by the median of overall accuracy, which exceeds 95%. The SCF helps to enhance runoff and snow simulations for 39% and 84% of the overall catchments in validation period, respectively. The use of SCF in model calibration improves the efficiency of runoff model, particularly in lowland catchments.

## Introduction

The snow and ice melt produce water for roughly one-fifth of the global population^1^. Snowmelt is a dominant source of annual runoff and groundwater recharge, which, as baseflow, supports streamflow, aquatic ecosystems, and vegetation growth during low-flow seasons^1–4^. Additionally, the snowpack is a key component of the hydrological balance in many regions worldwide, particularly in mountainous areas^5,6^. Monitoring and modeling snow accumulation and melting in these regions is particularly challenging due to the large spatial variability of snow characteristics and limited access to ground-based hydrological data. As an alternative to ground-based data, satellite observations are increasingly used in mountainous areas because of their high resolution and availability, regardless of terrain conditions^5^.

Over the past decade, various types of remotely sensed snow observations have been used to enhance hydrological simulations at the catchment scale. These include snow cover products with different spatial and temporal resolutions, such as those from MODIS, MSG-Seviri, Landsat, or Sentinel missions. For example, the most frequently used snow cover product from the Moderate Resolution Imaging Spectroradiometer (MODIS) is commonly applied for regional snow cover mapping, as it provides high temporal (up to a day) and spatial (500 m) resolution of observations. Several evaluations have demonstrated that the accuracy of satellite snow cover observations exceeds 90% and that satellite observations are consistent with other satellite-derived snow products and ground-based point measurements of snow depth^5,7–13^.

In recent years, remote-sensed snow observations have also been incorporated into streamflow simulations^2–4^. There are many studies evaluating the assimilation of satellite snow cover data into operational runoff simulations or calibration and validation of hydrological models^5–10,14^. The majority of these studies have reported that assimilating satellite snow data into hydrological models substantially enhances snow cover simulations without a significant reduction in the performance of the runoff model. For example, a study^14^ applied MODIS snow cover area data to operational runoff simulation and demonstrated that the assimilation of MODIS snow cover data using the particle filter method improves the simulation of snow-covered areas and, in some cases, the efficiency of streamflow simulations. Similar results have been obtained by assimilating MSG-Seviri into the calibration of hydrologic models^15,16,15^. compared two satellite snow cover products (MODIS, MSG-SEVIRI) for the calibration of two hydrologic models and demonstrated that, although MSG-SEVIRI allows for an effective reduction of clouds, both products are consistent in the estimation of snow cover area and the simulation of daily discharges^17^. applied snow cover data from AVHRR in the model calibration of six catchments in Kyrgyzstan and reported only small trade-offs between runoff efficiency and snow cover error. However, they found that snow cover errors may be large when snow cover is neglected during calibration. Recently^18^, evaluated different variants of multiple-objective calibration using satellite data of snow cover and soil moisture. They reported that satellite observations enable not only the improvement of internal consistency in conceptual hydrologic models, but also the improvement of simulations of daily runoff during the validation period.

A typical setup of hydrological models that uses only runoff is often associated with large parameter uncertainty, which impacts the reliability of hydrological simulations of other water cycle components^19^. Multiple-objective calibration, which integrates multiple observational data types, including those from remote sensing, offers a more robust setup and validation of hydrologic models. This is particularly important in mountainous areas with sparse in situ observations but large gradients of physiographic characteristics. The increasing availability of high-resolution satellite snow data enables further improvements in hydrologic model simulations, addressing the current limitations of using satellite products of snow, namely, high cloud coverage during winter and spring and misclassification of shaded areas in complex mountain terrains.

Recent observations from the Sentinel-3 multi-sensor platform offer significant advantages for snow monitoring due to its high spectral resolution and daily global coverage^20^. The Sentinel-3 mission retrieves important snow properties, such as broadband albedo, snow grain size, and fractional snow cover, making it particularly valuable for hydrological applications^21^. The data from the optical sensor (OLCI) and thermal radiometer (SLSTR) provide direct measurement of snow cover extent, enabling more comprehensive snow characterization using a spectral unmixing approach^22^. As the snow reflectance differs with illumination conditions, particularly in mountainous regions, the multispectral unmixing method accounts for this variability by adapting reference spectra locally, which results in reducing the misclassification of shaded snow. Thus, the value of such a product for hydrological modelling, however, still needs to be assessed.

The main objective of this study is to evaluate the potential of the new snow cover fraction (SCF) product from the Sentinel-3 mission for improving hydrological simulations at the regional scale. The specific aims are: (a) to compare the snow cover mapping accuracy with daily snow depth observations at 631 climate stations; (b) to assess and compare the runoff and snow model efficiencies obtained from a multiple-objective calibration and those from calibration to runoff only. The study evaluates and compares the snow and runoff performance of a conceptual hydrologic model in 188 lowland and alpine catchments, identifying whether and where the use of SCF improves hydrological simulations in Austria.

## Data and study area

### Snow cover fraction from Sentinel-3 satellite

Snow cover fraction (SCF) products derived from Sentinel-3 SLSTR and OLCI observations are generated using the Locally Adaptive Multi-Spectral Unmixing (LAMSU) algorithm^22^. In contrast to satellite products (such as MODIS), which map snow cover using a normalized difference snow index (NDSI) (i.e., an index that relates the high reflectance of snow in the visible part and the low reflectance of snow in the shortwave infrared part of the spectrum), the Sentinel-3 SCF product is derived based on a multispectral unmixing method. Basically, this approach assumes two types of endmembers, i.e., snow-free and snow-covered, for an observed spectrum and directly selects them from the pixels within the image (scene). Snow-free endmembers represent typical surface types, e.g., grass, sand, and rock, which differ across various regions. Snow-covered endmembers exhibit different snow spectra based on grain sizes^22^. This approach efficiently accounts for spatial variability in illumination conditions, which is particularly relevant in complex mountainous terrain. The method enables reliable SCF retrieval even in shaded areas by exploiting the full spectral range of both (OLCI and SLSTR) Sentinel-3 instruments, while leveraging the higher spatial resolution of OLCI for enhanced mapping accuracy. More details about the mapping approach are provided in^22^.

Daily SCF products used in the study are available for the period January 2017 to December 2023, covering the entire Alpine domain (50°N–43°N, 4°E–18°E) with a spatial resolution of 0.00200° × 0.00200°. Each product provides the proportion of snow-covered area per pixel, expressed as a percentage. In forested regions, the SCF represents the fraction of snow viewable from the sensor’s perspective.

Water bodies in SCF are masked using the Copernicus Global Surface Water dataset, applying the combined maximum extent of permanent and seasonal water bodies observed annually between 2017 and 2021^23^. Cloud pixels are identified and masked using an adapted version of the Simple Cloud Detection Algorithm^24^.

### **Hydroclimatic data**

**Fig. 1:**
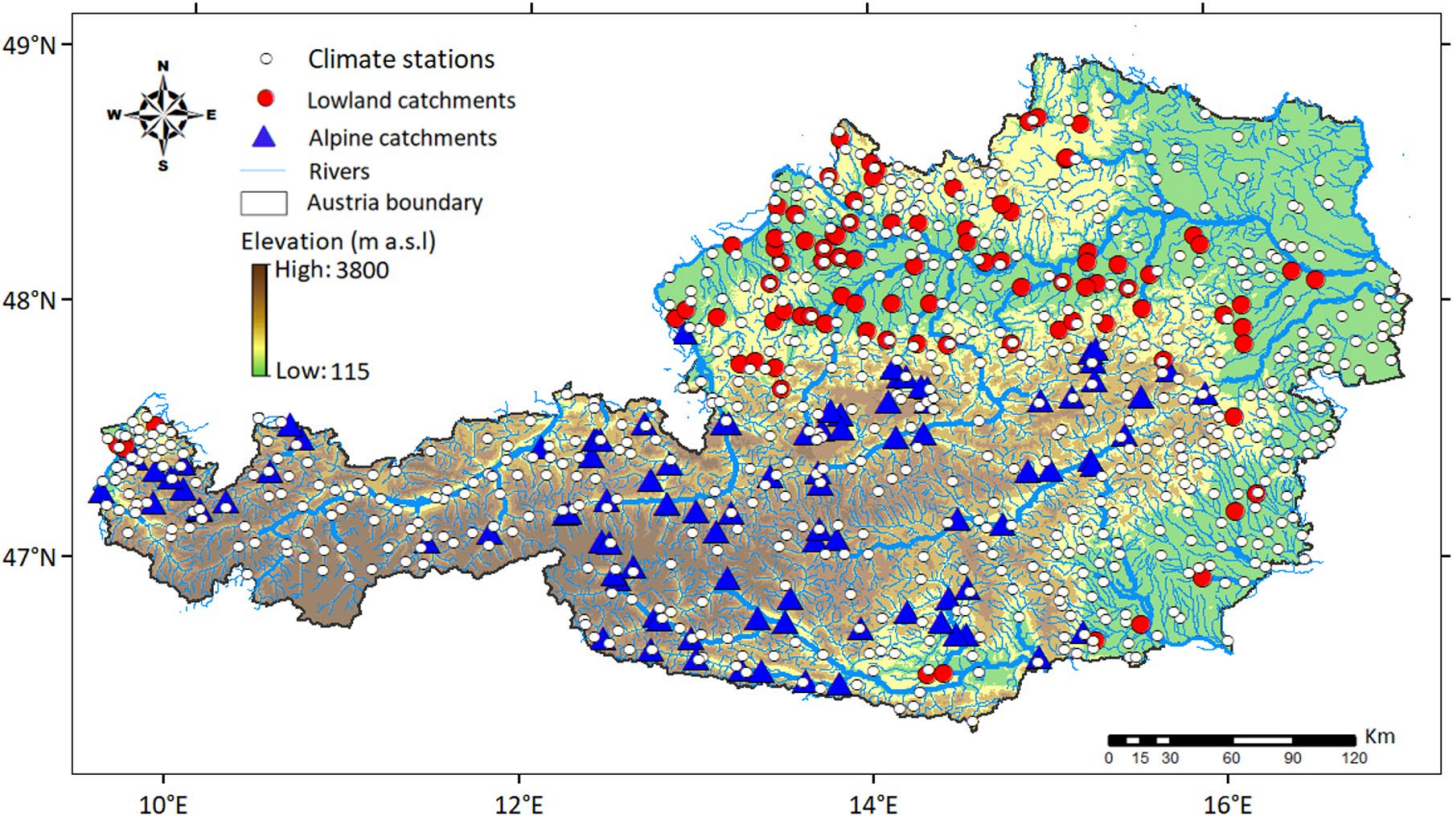
Topography of Austria alongside location of climate stations and gauges with daily runoff observations. Climate stations, lowland, and alpine catchments are displayed using white, red (lowland), and blue (alpine) symbols, respectively. The map is created in ArcMap 10.4 (Environmental Systems Resource Institute, ArcMap 10.4 ESRI, Redlands, California, USA; https://www.esri.com).

To evaluate the agreement between snow cover product and snow depth measurements, the SCF product is compared with daily snow depth measurements at 631 climate stations from January 2017 to December 2023, corresponding to the network used in previous studies^10,11,25^. For this purpose, the SCF product was extracted for the pixels where the climate stations are located. The snow depth observations are collected and available at the data portal of the Hydrographic Service of Austria (https://ehyd.gv.at/). Snow depth measurements represent point measurements taken at permanent staff gauges (permanently mounted snow stakes) at open locations, taken daily at 7:00 AM, and total snow depths are reported as integer centimeter values^25^. The spatial distribution of the climate stations (Fig. 1) covers a wide range of elevation zones in Austria. However, in the high alpine regions, the stations are typically located in the valleys at lower elevations^10,11,25^.

The inputs for hydrological modelling are obtained from the SPARTACUS database available at the data portal of Geosphere Austria (https://data.hub.geosphere.at/dataset/spartacus-v2-1d-1 km). The SPARTACUS database provides daily grid maps of precipitation and air temperature at a 1 km resolution for the entire territory of Austria^26,27^. Mean daily potential evaporation is derived from gridded maps of mean daily air temperature and potential sunshine duration index by using a modified Blaney–Criddle approach^28^.

The daily runoff observations for selected catchments are provided and available at the data portal of the Hydrographic Service of Austria (https://ehyd.gv.at, last access: 10/02/2025). All daily data for hydrological modelling are available for the period from 1 September 2017 to 31 August 2022.

### Study region

The study region is Austria. The area of Austria covers approximately 84,000 km². Austria is an ideal testbed for the evaluation of Sentinel-3 snow cover fraction at the regional scale. It is characterized by flat terrains in the eastern and northern parts, and alpine topography in the western and central parts (Fig. 1). Elevations vary from 115 m a.s.l. to 3800 m a.s.l. Climatologically, Austria is situated within a temperate climate zone, with mean annual precipitation of less than 400 mm yr⁻¹ in the lowlands and more than 2800 mm yr⁻¹ in the western Alps. Land use is predominantly agricultural in the lowlands and forested in the mid-elevation areas. The highest mountain regions are characterized by alpine vegetation and extensive rocky surfaces.

The potential of SCF for improving hydrological modelling is evaluated across 188 catchments (Fig. 1). The catchment sizes range from 13.7 to 6214 km². To evaluate the impact of elevation and climate on modelling performance, the analyzed catchments are split into two groups based on mean catchment elevation^29,30^: (1) 90 lowland catchments in drier lowland and hilly regions, (mean elevation below 900 m a.s.l.), and (2) 98 alpine catchments in wetter alpine regions, with a mean elevation exceeding 900 m a.s.l.

## Methods

### Agreement of Sentinel-3 Snow Cover Fraction and daily snow depth data at climate stations

The SCF product has been compared with daily snow depth observations at climate stations. The consistency of both datasets has been quantified by the overall accuracy index ($\:{O}_{A}$) as below:

1 $$\:{O}_{A}=\frac{a+d}{a+b+c+d}\cdot\:100$$

where $\:a$, $\:b$, $\:c$, and $\:d$ represent the frequencies of agreement and disagreement between remotely sensed SCF and in-situ snow depth observations at climate stations, as defined in Table 1^25^. The $\:{O}_{A}\:$indicates a metric of the relative frequency of days with agreement between the SCF extracted for the pixel representing the location of the climate stations and snow depth measurements at that station, related to the total number of days with observations.

**Table 1:** Definition of agreement and disagreement frequencies between Sentinel-3 snow cover fraction product (SCF) and snow depth measurements at climate stations. The $\:a$, $\:b$, $\:c$, and $\:d$ frequencies are used to estimate the overall accuracy (Eq. 1) and SCF over- and under-estimation errors (Eqs. 2 and 3).

Frequency	SCF > 0%	SCF = 0%
Station snow depth > 0 cm	$\:a$	$\:b$
Station snow depth = 0 cm	$\:c$	$\:d$

In a similar way, the SCF over- ($\:{O}_{E}$) and under-estimation ($\:{U}_{E}$) errors have been determined. The $\:{O}_{E}$ and $\:{U}_{E}$ are defined as:

2 $$\:{O}_{E}=\frac{c}{a+b+c+d}\cdot\:100$$

3 $$\:{U}_{E}=\frac{b}{a+b+c+d}\cdot\:100$$

the $\:{O}_{A}$, $\:{O}_{E}$ and $\:{U}_{E}$ have been estimated for individual stations and whole period of observation (2017–2023) as well as monthly for groups of stations located in different elevation zones. In this case, the $\:a$, $\:b$, $\:c$, and $\:d$ (Table 1) represent the frequencies of station-days for a given time period (i.e., month). It is worth noting that there is no specific standard to determine a threshold for no snow in the SCF product. Given that the frequency of days with no snow at the climate stations (snow depth = 0 cm) and a snow cover fraction between 1% and 5% (1 ≤ SCF < 6) is negligible (around 1.2% of station-days cloud free) during 2017–2023, it has no significant impact on overall accuracy.

### Evaluation of Sentinel-3 snow cover fraction for improving hydrological simulations

#### Conceptual hydrologic model

The potential of the Sentinel-3 SCF product for improving hydrological simulations is tested using a conceptual hydrological TUW model^31–33^. The TUW model has been used in numerous studies in the study region in the past^5,30,32,34–36^. Thus, it allows a direct comparison of model efficiencies with previous results.

The model consists of three routines: snow accumulation and melt, soil moisture accounting, and runoff routing. In total, it has 15 model parameters (Table 2), which are estimated in model calibration. The snow module has five model parameters: the snow correction factor (SCF), which accounts for errors in measuring snowfall due to gauge undercatch; the degree–day factor (DDF); and three threshold temperatures (Ts, Tr, and Tm) that control the form of precipitation and melt. The soil moisture routine has three parameters: the maximum soil moisture storage in the root zone field capacity (FC), a limit that controls actual evapotranspiration relative to potential evapotranspiration (LP), and a nonlinear parameter for runoff production (Beta). The simulation of the routing process involves two parts, i.e., within-catchment routing and stream routing. The within-catchment routing has five parameters: i.e., three storage coefficients (K_0_, K_1_, and K_2_) that control overland flow, interflow, and base flow, respectively; a threshold for very fast response (Lsuz); and a constant percolation rate (Cperc) connecting the fast and slow reservoirs. The stream routing uses a triangular transfer function with two parameters (Bmax and Croute) for routing runoff.

The model is used in a semi-distributed way, i.e., model inputs and outputs are estimated for elevation zones of 200 m, while the model parameters are assumed to be constant in each catchment. More details about the model can be found in^32,37^.

**Table 2:** List of TUW model parameters and their range used in automatic model calibration.

Sub-Routines	Model parameters	Explanation (unit)	Range used in calibration
Snow	SCF	Factor for correcting snow measurements [-]	0.9–1.5
	DDF	Degree-day factor determines the speed of the snow melting [mm/°C/day]	0–5
	Tr	Temperature threshold above which precipitation is liquid [°C]	1–3
	Ts	Temperature threshold below which precipitation is solid [°C]	-3–1
	Tm	Temperature threshold above which snowmelt starts [°C]	−2–2
Soil moisture	LP	A limit for potential evapotranspiration [-]	0–1
	FC	Field capacity- maximum soil moisture storage [mm]	0–600
	Beta	Coefficient influencing the amount of water caused by soil moisture and the upper reservoir [-]	0–20
Runoff response	K_0_	The storage coefficients associated with the surface (K_0_), sub-surface (K_1_), and base flow (K_2_) [days]	0–2
	K_1_		2–30
	K_2_		30–250
	Lsuz	Threshold storage state for initiating very fast surface runoff [mm]	1–100
	Cperc	Constant percolation rate from the upper to the bottom reservoir [mm/day]	0–8
	Bmax	Maximum base parameter [day]	0–30
	Croute	Free scaling parameter [day^2^/mm]	0–50

#### Calibration and validation of the conceptual hydrologic model

The approach used for assessing the impact of using satellite SCF in hydrological model calibration and validation follows the strategy presented in^18^. The hydrologic model is calibrated using two variants: (1) the traditional calibration to runoff only (*CALQ*); (2) the multiple-objective calibration (*CALM*) to runoff and satellite SCF. The general form of the calibration objective function ($\:F$) consists of maximizing the weighted sum of runoff ($\:{O}_{Q}$) and snow cover fraction ($\:{O}_{SCF}$) functions as follows:

4 $$\:F=\:{w}_{Q}.{O}_{Q}+\:{w}_{SCF}{.O}_{SCF}$$

where $\:{w}_{Q}$ and $\:{w}_{SCF}$ are the weights of the runoff and SCF objective functions. While the *CALQ* calibration variant assumes $\:{w}_{Q}=1$ and $\:{w}_{SCF}=0$, the *CALM* calibration variant uses equal weights for runoff and SCF objective functions, i.e., $\:{\mathrm{w}}_{\mathrm{Q}}=0.5$ and $\:{\mathrm{w}}_{\mathrm{S}\mathrm{C}\mathrm{F}}=0.5$. This choice is based on previous studies^18,38^, who also found that the runoff weight coefficients greater than 0.3 yields robust calibration results.

The individual objectives $\:{O}_{Q}$ and $\:{O}_{SCF}$ are defined in Eq. 5 and Eq. 8. The runoff objective $\:{O}_{Q}$ consists of a combination of two variants of the Nash–Sutcliffe efficiency coefficient, $\:NSE$ and $\:{NSE}_{log}$^39^, as follows:

5 $$\:{O}_{Q}=\left(0.5\cdot\:NSE+0.5\cdot\:{NSE}_{log}\right)$$

6 $$\:NSE=1-\frac{\sum\:_{i=1}^{n}{\left({\mathrm{Q}}_{\mathrm{s}}-{\mathrm{Q}}_{\mathrm{o}}\right)}^{2}}{\sum\:_{i=1}^{n}{\left({\mathrm{Q}}_{\mathrm{o}}-{\stackrel{-}{\mathrm{O}}}_{\mathrm{o}}\right)}^{2}}$$

7 $$\:{NSE}_{\mathrm{l}\mathrm{o}\mathrm{g}}=1-\frac{\sum\:{\left({\mathrm{l}\mathrm{o}\mathrm{g}(\mathrm{Q}}_{\mathrm{s}})-{\mathrm{l}\mathrm{o}\mathrm{g}(\mathrm{Q}}_{\mathrm{o}}\right))}^{2}}{\sum\:{\left(\mathrm{l}\mathrm{o}\mathrm{g}\left({\mathrm{Q}}_{\mathrm{o}}\right)-\mathrm{l}\mathrm{o}\mathrm{g}\left({\stackrel{-}{\mathrm{Q}}}_{\mathrm{o}}\right)\right)}^{2}}$$

where $\:{Q}_{s}$ and $\:{Q}_{o}$ are the mean daily simulated and observed runoff, respectively, and $\:{\stackrel{-}{Q}}_{o}$ is the average of observed daily runoff over the calibration (or verification) period of $\:n$ days. The choice of using a combination of $\:NSE$ and $\:{NSE}_{log}$ is based on a previous study^18^, which enables weighting both high- and low-flow conditions during model calibration.

The SCF objective function $\:{O}_{SCF}$ minimizes the relative number of days with poor snow cover simulations. The $\:{O}_{SCF}\:$sums the snow over- ($\:{S}_{O}$) and under- ($\:{S}_{U}$) estimation errors and relates it to the number of days ($\:nc$) with lower cloud coverage in satellite SCF (i.e., mean catchment cloud coverage less than 60%):

8 $$\:{O}_{SCF}=1-\left(\frac{({S}_{O}+{S}_{U})}{nc}\right)$$

The model snow underestimation error ($\:{S}_{U}$) calculates the weighted sum of days for which the hydrologic model fails to simulate snow cover. The model snow overestimation error ($\:{S}_{O}$) refers to the weighted sum of days for which the hydrologic model simulates snow, while the satellite does not observe snow cover. The thresholds used in the definition of snow-related errors are 10 mm for the hydrologic model and 50% for the satellite product^5,18^. These values were selected based on sensitivity tests in prior studies^5,18^. Basically, to achieve a balance between computational efficiency and the practical need for catchment-scale streamflow simulation, the SCF product was aggregated to the catchment scale for both calibration and validation. Consequently, the snow objective function utilizes lumped catchment-mean snow information to align with the semi-distributed model representation. Notably, the higher the runoff and snow model efficiencies ($\:{O}_{Q}$ and $\:{O}_{SCF}$), the better model performance.

Model parameters are calibrated automatically in each catchment and for each calibration variant, *CALQ* and *CALM*. Automatic calibration is based on the DEoptim calibration package implemented in the R software^40^. In both calibration variants, the hydrologic model is calibrated from September 1, 2017, to August 31, 2020, and validated from September 1, 2020, to August 31, 2022. The model warm-up period is 365 days prior to the start of the calibration or validation period.

## Results

### Agreement of Sentinel-3 snow cover fraction and daily snow depth data at climate stations

**Fig. 2:**
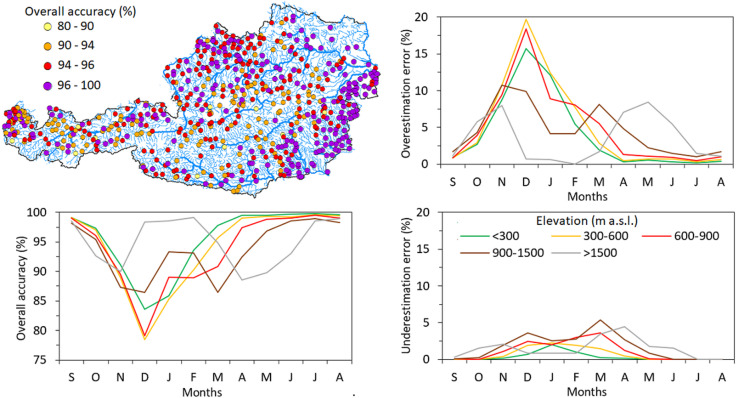
The agreement between Sentinel-3 snow cover fraction and daily snow depth observations at 631 climate stations in the period 2017–2023. The overall accuracy (left panels) shows the spatial and temporal variability across climate stations situated in different elevation zones. The right panels show the temporal variability of over- and underestimation errors expressed as median of disagreement at climate stations situated in different elevation zones. The map is created in ArcMap 10.4 (Environmental Systems Resource Institute, ArcMap 10.4 ESRI, Redlands, California, USA; https://www.esri.com).

The overall accuracy ($\:{O}_{A}$) between satellite SCF and snow depth measurements at 631 climate stations is presented in Fig. 2. While the map (top left panel) shows the spatial variability of $\:{O}_{A}$ of individual stations, the line graphs show the monthly agreement $\:{O}_{A}$ (Fig. 2 bottom left panel) and the SCF over- (top right panel) and under- (bottom right panel) estimation errors for stations grouped into five elevation zones. The results show that the agreement between satellite SCF and snow depth observations at climate stations is very high, but varies in different months. The median $\:{O}_{A}$ obtained from 631 climate stations during the entire period (2017–2023) exceeds 95.3%, with an interquartile range (IQR) of 2.65%.

The monthly $\:{O}_{A}$ varies by elevation zone. While for the winter months (December-February), the median agreement is for stations located above 1500 m a.s.l. larger than 98%, for the spring months (March-June), the agreement drops to 90–95%. For lower elevations (stations located below 900 m a.s.l.), the largest disagreement is observed during the winter months, where, for example, in December, the median $\:{O}_{A}$ drops below 80%. The comparison of over-(*O*_*E*_) and under- (*U*_*E*_) estimation errors indicates that the overestimation errors are higher than the underestimation errors, meaning SCF more frequently shows snow cover when snow depth is zero at climate stations than the opposite state. Interestingly, while the *O*_*E*_ is the largest for lower elevations in December, in alpine regions (i.e., stations above 1500 m a.s.l.), the *O*_*E*_ is the largest in November and May. The *U*_*E*_ errors are below 5% in all elevation zones and months.

**Fig. 3:**
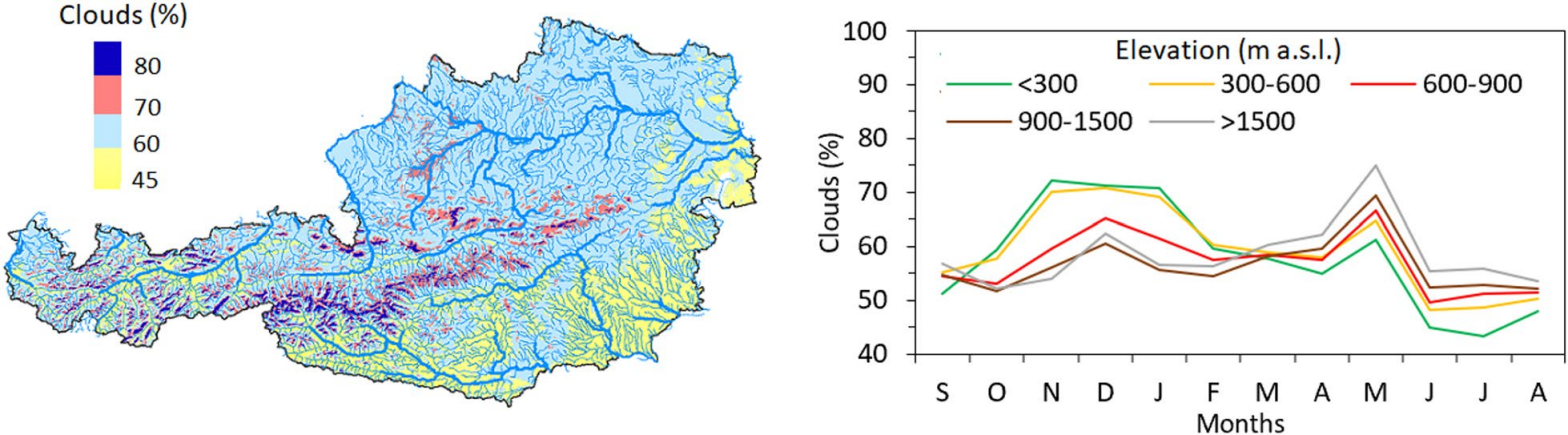
Frequency of pixels cloud covered in Sentinel-3 snow cover fraction product in Austria. The map (left panel) shows the mean cloud coverage in the period 2017–2023, the line graph (right panel) shows the monthly variability of median cloud coverage at climate stations in different elevation zones. The map is created in ArcMap 10.4 (Environmental Systems Resource Institute, ArcMap 10.4 ESRI, Redlands, California, USA; https://www.esri.com).

The evaluation of the agreement between SCF and snow depth observations at climate stations is linked with the estimated frequency of pixels cloud covered. Figure 3 shows the spatial and temporal variability of cloud coverage over Austria in the period 2017–2023. The results indicate that the mean cloud coverage over Austria is 57.8%. The median cloud coverage is the largest at lower elevation stations (below 600 m a.s.l.) from November to January, and at elevations above 1500 m a.s.l. in May when it exceeds 70%. There is an interesting switch in the dependence of cloud coverage on elevation in February and March. While cloud cover increases with elevation from April to August, lower elevations have more clouds than alpine locations above 1500 m a.s.l. between October and February. This indicates that the SCF product shows more cloud coverage for lowland areas during the winter months than for mountains.

### Evaluation of Sentinel-3 snow cover fraction for improving hydrological simulations

**Fig. 4:**
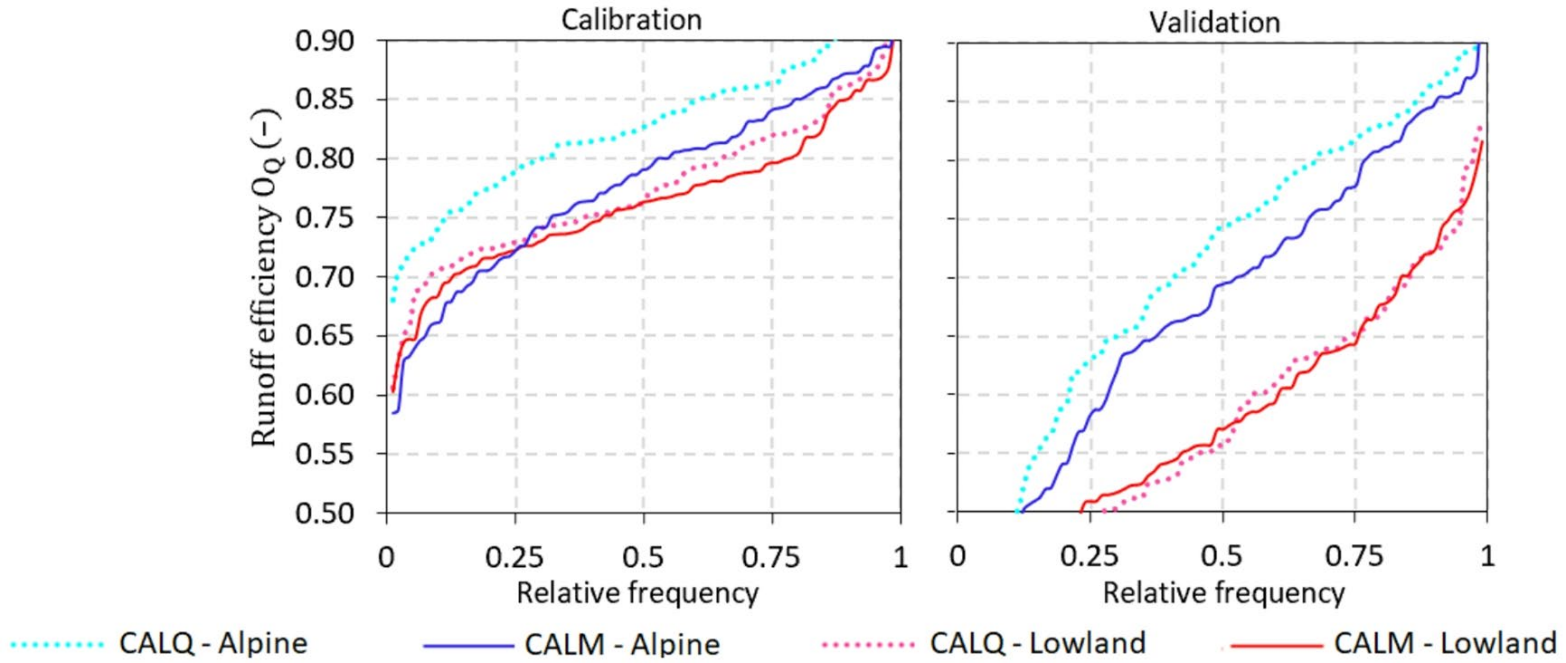
Cumulative distribution functions of the runoff model efficiencies (*O*_*Q*_, Eq. 5) for two calibration variants (i.e., runoff-only *CALQ*, and multiple-objective calibration using snow cover fraction *CALM*) in the calibration (left panel) and validation (right panel) periods. Blue and red lines represent the *O*_*Q*_ for the alpine and lowland catchments, respectively.

**Fig. 5:**
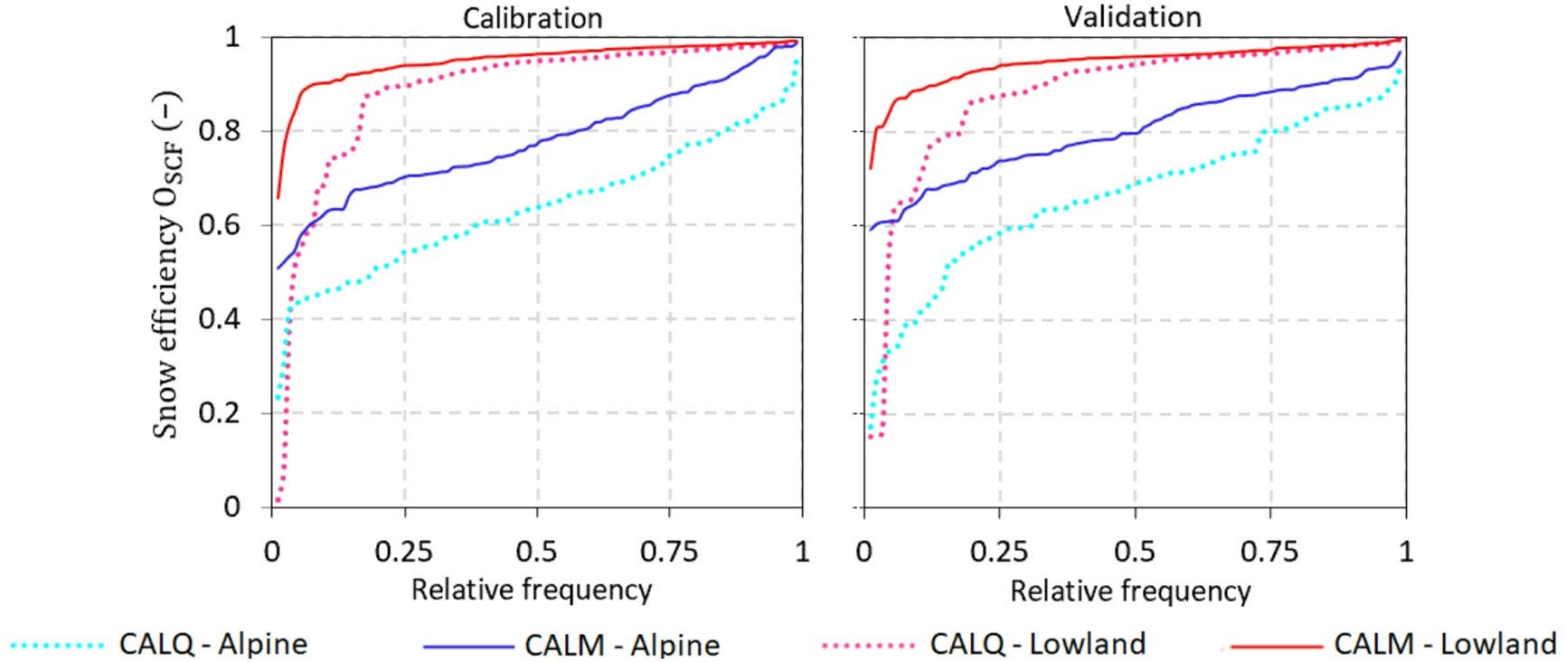
Cumulative distribution functions of the snow model efficiencies (*O*_*SCF*_, Eq. 8) for two calibration variants (i.e., runoff-only *CALQ*, and multiple-objective calibration using snow cover fraction *CALM*) in the calibration (left panel) and validation (right panel) periods. Blue and red lines represent the *O*_*SCF*_ for the alpine and lowland catchments, respectively.

**Fig. 6:**
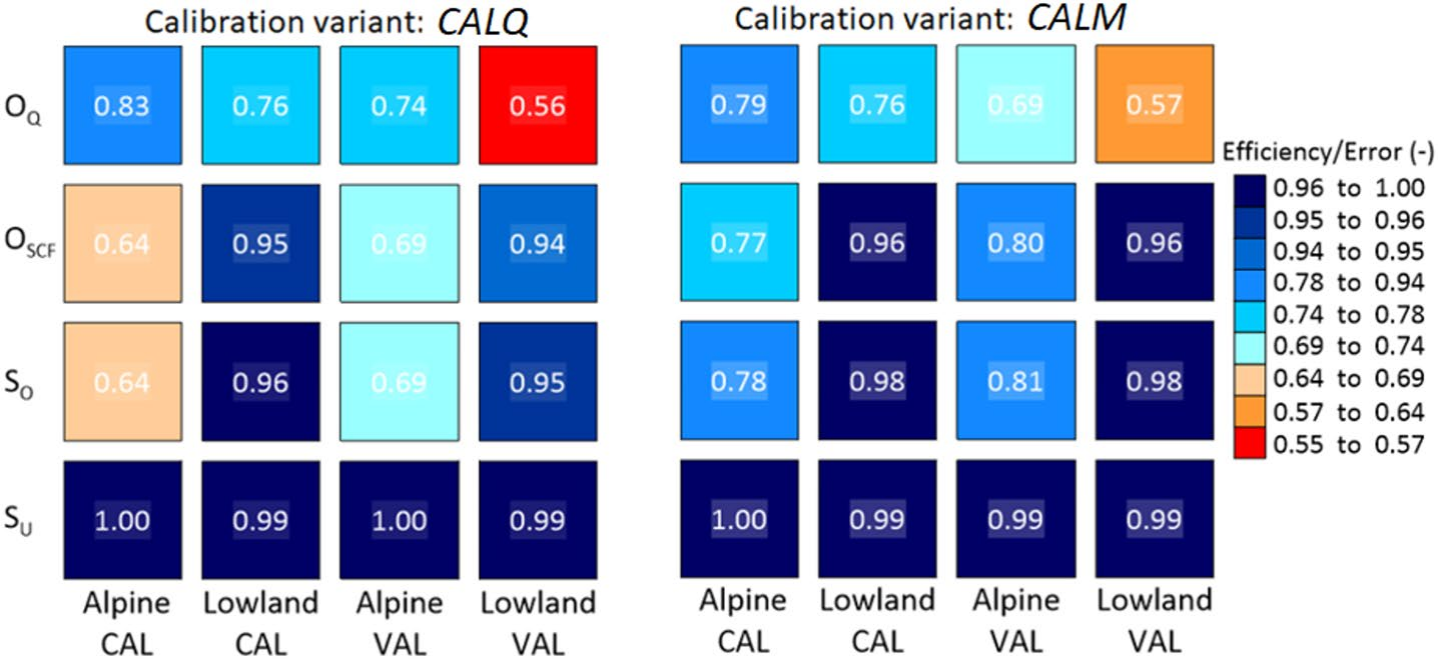
Median of the calibration (CAL) and validation (VAL) runoff (*O*_*Q*_) and snow (*O*_*SCF*_, *S*_*O*_, *S*_*U*_) model efficiencies obtained for two calibration variants (runoff only-*CALQ* and multiple-objective calibration-*CALM*) in the alpine and lowland catchments. Runoff and snow efficiencies, as well as snow over- and under-estimation errors, are defined in Sect.  3.2.2.

Runoff and snow model efficiencies and their medians for the calibration and validation periods under both calibration variants (*CALQ* and *CALM*) are presented in Figs. 4, 5 and 6. Figure 4 shows the cumulative distribution functions of runoff model efficiency (*O*_*Q*_) for alpine and lowland catchments in the calibration (left panel) and validation (right panel) periods. The results show that the difference in runoff model efficiency is mainly observed in alpine catchments based on both calibration variants. In the calibration period, the median runoff model efficiency in alpine catchments is 4% higher for *CALQ* than for *CALM* (Fig. 6). In the lowland catchments, both variants have very similar runoff model efficiencies in the calibration period. The median for both calibration variants (*CALQ* and *CALM*) is 0.76 in lowland catchments. Similarly, in the validation period (Fig. 4, right panel), the runoff-only variant (*CALQ*) has a larger runoff model efficiency in alpine catchments. Interestingly, in the lowland group of catchments, the median of runoff efficiency is slightly larger for the *CALM* variant. This suggests that using snow cover fraction data enhances the efficiency of the runoff model in numerous lowland catchments.

The snow model efficiency for both variants is presented in Fig. 5. Using SCF data in model calibration results in better snow model performance for the *CALM* variant in both alpine and lowland catchments. In the alpine catchments, the snow model efficiency is remarkably higher; for example, the median of $\:{O}_{SCF}$ in the calibration and validation periods (Fig. 6) is approximately 13% and 11% higher for *CALM* compared to *CALQ*, respectively. Figure 6 summarizes also the type of snow error, i.e., the snow overestimation ($\:{S}_{O}$) and underestimation ($\:{S}_{U}$) errors. The results indicate that the hydrologic model tends to overestimate SCF, particularly in alpine catchments. Accordingly, the median of overestimation for the *CALM* variant is approximately 14% and 12% larger than for the *CALQ* variant in the calibration and validation period, respectively. In the lowland catchments, the median snow overestimation errors are only about 2–3% larger for the *CALM* than for the *CALQ*.

**Fig. 7:**
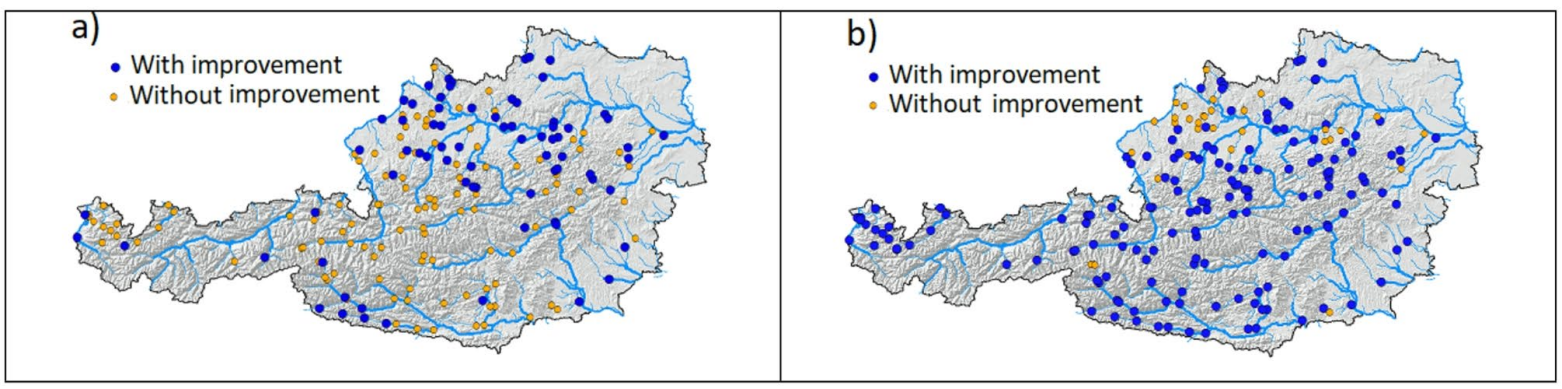
Location of gauges (blue circles) in which multiple objective calibration (*CALM*) outperforms runoff (left panel) and snow (right panel) simulations of runoff-only (*CALQ*) calibration in the validation period 2020–2022. Orange circles show gauges where *CALQ* efficiency is higher than that of *CALM*. The map is created in ArcMap 10.4 (Environmental Systems Resource Institute, ArcMap 10.4 ESRI, Redlands, California, USA; https://www.esri.com).

Figure 7 shows the spatial pattern of gauges where using SCF in model calibration (i.e., *CALM* calibration variant) improves simulations of the hydrologic model in the validation period. Blue symbols indicate stations where the validation runoff (left panel) and snow (right panel) efficiency is larger for *CALM* than for *CALQ*. Table 3 summarizes the number of gauges with improvements for both groups of catchments. The results show that the use of SCF in *CALM* improves runoff simulations, i.e., the runoff model efficiency, particularly in lowland catchments. While in the alpine catchments, runoff improvement is found in 24% of catchments, in the lowlands, it is improved in 54% of catchments. In contrast, the snow simulations are improved in 98% of alpine and only in 68% of lowland catchments, respectively.

**Table 3:** The number of catchments in which the multiple-objective calibration (*CALM*) outperforms runoff and snow model simulations obtained by the runoff-only calibration variant (*CALQ*) in the validation period (2020–2022).

Calibration variant	Alpine (98)		Lowland (90)	
	No.	(%)	No.	(%)
$\:{O}_{Q}$	24	24	49	54
$\:{O}_{SCF}$	96	98	61	68

## Discussion and conclusions

This study evaluates the potential of the new Sentinel-3 snow cover fraction product (SCF) for improving hydrological simulations at the regional scale. Compared to alternative satellite snow cover products, which classify snow cover using an NDSI index, the SCF estimates snow cover fraction using a multispectral unmixing method, in which endmember spectra are extracted directly from the image (scene)^22^. Such an approach allows accounting for locally variable illumination conditions in complex mountainous terrain and thereby reduces the classification errors caused by spatially heterogeneous illumination conditions. The results indicate that this alternative mapping approach reduces the frequency of cloud mapping, particularly in alpine regions in winter months, thereby reducing errors in misclassifying snow as cloud. Previous assessments of MODIS snow cover products over Austria indicated substantial cloud coverage over 80% in December and January^10,11,25^, whereas the SCF cloud coverage used in this research is below 60% for locations above 900 m a.s.l. While conservative masking of clouds in alpine terrain in NDSI-based products can significantly limit snow cover observations in the mountains, the new SCF provides an attractive source of snow cover estimates during the entire snow accumulation and melt seasons.

The assessment of SCF mapping accuracy at the regional scale shows a very good snow cover mapping accuracy. The overall accuracy exceeds 95%, which compares very well with similar evaluations of MODIS products in the study region; for example^10^, reported the same overall accuracy when evaluating one of the previous versions of the daily MODIS product over Austria. Recently^11^, found that when NDSI is optimized across different seasons and altitudinal zones, the mean overall accuracy of MODIS can exceed 97%. However, if we exclude cases with potential patchy snow cover where no snow depth can be observed at the climate stations, and SCF is below 10%, the overall accuracy increases to 97%. The advantage of mapping snow cover fractions within the pixel scale cannot be fully explored by using snow depth observations at climate stations; however, several alternative datasets and methodological approaches could provide a more comprehensive assessment. In light of this, comparison of the Sentinel-3 SCF product with high-resolution satellite data from Landsat-8 OLI (30 m spatial resolution and16-day temporal resolution) and Sentinel-2 MSI (20 m pixel spacing and 5 days repeat pass cycle) can be beneficial for the accuracy assessment of subpixel snow fraction estimates across diverse mountain regions^22,41,42^. Additionally, advanced methods for fractional snow cover retrieval, incorporating explicit error propagation frameworks, yield reliable benchmark datasets for validation^22^. The advantage of the SCF product for mapping snow cover in mountains is clearly found at the regional scale. The assessment of snow cover mapping errors showed that snow cover overestimation errors are generally larger than underestimation errors. Interestingly, in higher elevations, these overestimation errors are significantly lower than those reported in earlier accuracy assessments. For example^25^, reported that the mean overestimation errors at stations above 1500 m a.s.l. were larger than 10% from December to March, whereas the SCF overestimates snow cover in higher locations by less than 3% in those months.

The new snow cover fraction product derived from Sentinel-3 observations demonstrated a clear potential for enhancing hydrological simulations. In general, the runoff model efficiency obtained from multiple-objective calibration to runoff and SCF is fully within the range of previous assessments in Austria and similar physiographic regions. A direct comparison is not possible, as calibration and validation periods or number of catchments differ between the studies; however, the median runoff efficiency of 0.80 for the *CALQ* and 0.77 for the *CALM* calibration variants in the calibration period across all catchments found in this study are consistent with the results of earlier studies^5,18,32,43^. Majority of previous studies in other regions have reported, as the main benefits of using satellite snow cover data in hydrological modeling, an improvement in the consistency of model states^17,44^ or a reduction in hydrological modeling uncertainty^45^. We found that using new Sentinel-based SCF in model calibration improves not only snow but also runoff simulations in the validation period. The multiple-objective calibration has overall improved runoff and snow simulations in 39% and 84% of catchments, respectively. The analysis showed that most of the catchments with runoff improvement are situated at lower elevations, i.e., having the mean catchment elevation below 900 m^22^. compared the LAMSU method against very-high-resolution SCF maps from WorldView-2/3 data and revealed that the SCF retrieval errors increase from 14.3% to 21.4% in complex and shaded terrain. Consequently, the SCF product is likely more applicable to relatively homogeneous low elevation catchments than to highly heterogeneous alpine terrain, where mixed illumination and surface conditions produce strong sub-pixel variability. In addition, previous studies have shown that the improvement in runoff model efficiency using snow products tends to be small^46,47^. As the semi-distributed structure of the hydrological model showed high runoff model efficiency for the alpine catchments without snow data assimilation (see Fig. 6), incorporating the snow product provided limited additional benefit in these areas compared to the lowland catchments. Similar findings are also reported in^18^, who used a similar calibration concept with MODIS snow cover data. They found improvement in runoff model efficiency for 5% to 40% of catchments, depending on the weight given to snow or soil moisture satellite data in model calibration.

**Fig. 8:**
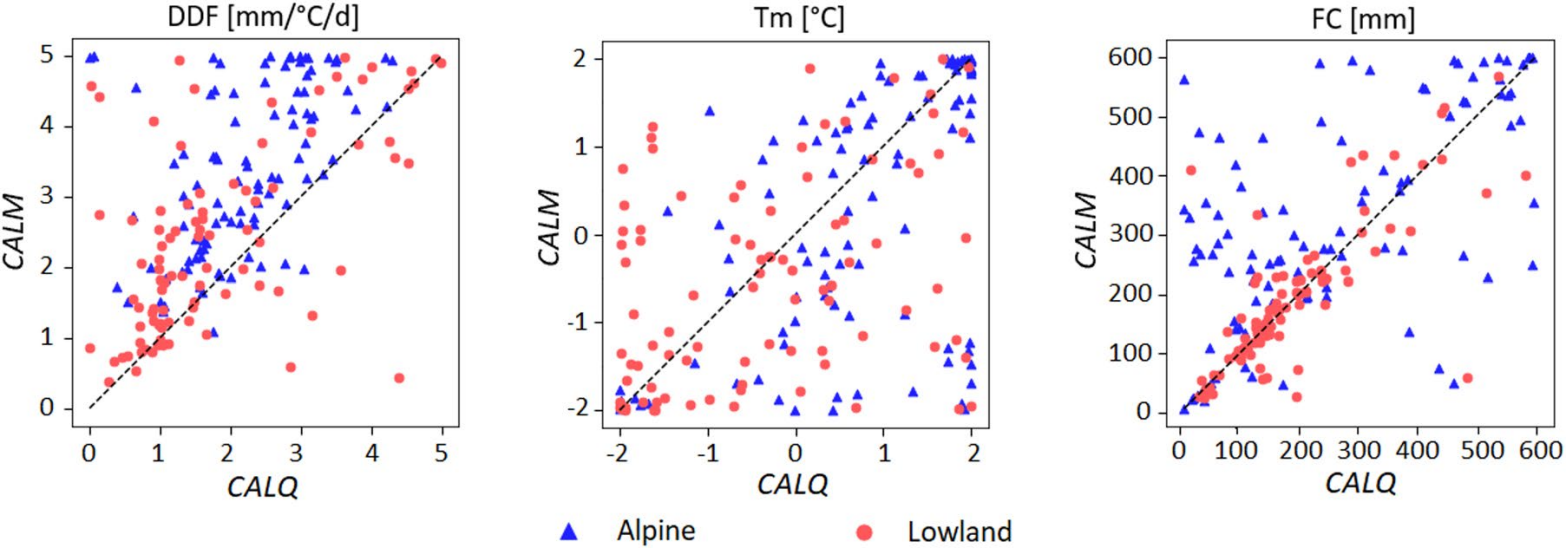
Comparison of model parameters (DDF, T_m_, and FC) calibrated to runoff only (*CALQ*) and to runoff and satellite snow cover fraction (*CALM*). The symbols indicate two groups of catchments, i.e., alpine (blue symbols) and lowland (red symbols) catchments.

The results of^18^ indicate that satellite snow cover has a particular impact on model parameters related to snow accumulation and melt processes. Figure 8 illustrates an example of the difference between selected model parameters calibrated using runoff alone and those calibrated using both SCF and runoff. The greater the dispersion of symbols around the 1:1 line (i.e., low correlation coefficient), the more strongly the parameters are influenced by the *CALM* compared to the *CALQ* calibration variant. Figure 8 clearly indicates that the impact of SCF differs not only for model parameters but also between alpine and lowland catchments. Although the threshold temperature above which snowmelt starts (T_m_) has low correlation coefficient of 0.54 and 0.38 for alpine and lowland catchments, respectively, the degree-day factor (DDF) shows a lower correlation coefficient of 0.51 for alpine catchments. Similar results was found in^18^. The comparison of correlations between model parameters obtained by runoff-only and multiple-objective calibration, which uses MODIS snow cover (with an equal weight as runoff), showed that snow model parameters, such as the degree-day factor (DDF) or threshold temperatures (T_m_, T_r_, T_S_), differ between the calibration variants (i.e., are only weakly correlated). Interestingly, our results suggest that using snow cover fraction data has a large impact on soil field capacity (FC) model parameter, mainly in alpine catchments. As indicated in^48^, the degree-day factor and field capacity are two of the most sensitive model parameters in terms of temporal stability^49^. Using satellite SCF data can thus improve and have a significant impact on hydrologic simulations and uncertainty evaluations connected with assessment of potential impacts of climate change on water resources.

We evaluated the accuracy of the SCF product based on snow depth observations at climate stations. Future evaluations of the product in regions with specific setups and data (e.g., along transects or using camera observations) will allow to provide further insights into the mapping accuracy of the product under different land cover types, aspects, or slopes. Moreover, shallow snowpacks can be one of the factors controlling mapping accuracy during the last phases of snowmelt, e.g., as shown in^50^. It would be interesting to test this in future development of the SCF product, particularly in regions with dedicated monitoring of snow cover patterns such as in^51^.

The results demonstrate that the use of Sentinel-3 snow cover fraction in hydrological modeling can improve the representation of hydrological fluxes and the prediction of runoff hydrographs at the catchment scale. This study focuses on the initial assessment of its availability, accuracy, and consistency at the regional scale. It is recommended to compare this product with the widely used MODIS snow cover data in future studies. Although the semi-distributed version of the TUW model applied in this study accounts for spatial variability of the model inputs, e.g., precipitation and temperature, by interpolating them into 200 m elevation zones, the aggregation of fine resolution (200 m) SCF data to the catchment scale may smooth out spatial snow heterogeneity, particularly in large catchments (> 6000 km²). Future studies will focus on further testing the value of snow cover fraction at the pixel scale, which can improve the snow cover representations in alpine catchments with large spatial gradients in topography, slopes or aspects. A more detailed assessment of snow cover fraction dynamics can be carried out using distributed hydrologic models, which are able to account for within grid variability of snow cover. Additionally, this study applied constant weight coefficients for runoff and snow objective functions based on recent studies^18,38^. The sensitivity analysis of the weight coefficients was performed using the MODIS snow cover data in a recent study^18^. The results revealed that there are no significant changes in hydrological model efficiencies when the weight coefficients are varied. Sensitivity analysis of the weight coefficients for a recently developed SCF product falls beyond the scope of this study and is left as a direction for future studies.

## Data Availability

The snow depth observations are available at the data portal of the Hydrographic Service of Austria (https://ehyd.gv.at/, accessed October 2025). The precipitation and temperature are collected from the SPARTACUS database available at the data portal of Geosphere Austria (https://data.hub.geosphere.at/dataset/spartacus-v2-1d-1 km, accessed on 10 February 2025). The daily runoff observations are available at the data portal of the Hydrographic Service of Austria (https://ehyd.gv.at, accessed on 10 February 2025). The Sentinel-3 SLSTR/OLCI data are provided on the Copernicus Open Access Hub (https://scihub.copernicus.eu/, accessed on 14 July 2022).
